# Molecular insights and integrated management of Glomerella leaf spot in apple

**DOI:** 10.1093/hr/uhag156

**Published:** 2026-04-17

**Authors:** Mati Ur Rahman, Suhua Bai, Shukhrat Abdurakhmonovich Khamdullaev, Ilkham Sarsenbay uly Aytenov, Hongmin Hou, Khurshid Sadullaevich Turakulov, Chaohua Dong, Yugang Zhang

**Affiliations:** College of Horticulture, Qingdao Agricultural University, Qingdao 266109, China; Engineering Laboratory of Genetic Improvement of Horticultural Crops of Shandong Province, Qingdao Agricultural University, Qingdao 266109, China; College of Life Sciences, Qingdao Agricultural University, Qingdao 266109, China; Institute of Genetics and Plant Experimental Biology, Academy of Sciences of Uzbekistan, Tashkent 111226, Uzbekistan; Institute of Genetics and Plant Experimental Biology, Academy of Sciences of Uzbekistan, Tashkent 111226, Uzbekistan; College of Horticulture, Qingdao Agricultural University, Qingdao 266109, China; Engineering Laboratory of Genetic Improvement of Horticultural Crops of Shandong Province, Qingdao Agricultural University, Qingdao 266109, China; Institute of Genetics and Plant Experimental Biology, Academy of Sciences of Uzbekistan, Tashkent 111226, Uzbekistan; College of Life Sciences, Qingdao Agricultural University, Qingdao 266109, China; College of Horticulture, Qingdao Agricultural University, Qingdao 266109, China; Engineering Laboratory of Genetic Improvement of Horticultural Crops of Shandong Province, Qingdao Agricultural University, Qingdao 266109, China

## Abstract

Apple (*Malus × domestica*) is one of the world’s premier fruit crops. Apple Glomerella leaf spot (GLS) is caused by *Colletotrichum* spp., jeopardizing global apple supply and incurring millions of dollars in annual losses. The development of resistant apple varieties has evolved from conventional selection breeding to deliberate breeding strategies that incorporate statistical analysis and experimental design, and now increasingly integrate genetic and genomic data. Innovative methods like marker-assisted selection and genetic editing have facilitated the development of cultivars resistant to GLS. Novel breeding techniques (NBTs), particularly genome editing, are increasingly vital for the precise and rapid development of disease-resistant crops. The CRISPR/Cas9 system is a powerful tool for targeted mutagenesis, enabling the development of genetically edited crops that are not classified as transgenic. Consequently, researchers are working to identify genes associated with susceptibility and resistance. The article explores the established mechanisms of interaction between the GLS pathogen and its host, as well as the innate immune responses elicited in the apple plant, to expedite the development of resistant apple varieties. Recent advances in effector biology and host gene targeting offer new avenues to enhance immunity and engineer durable resistance traits against GLS.

## Graphical Abstract

**Graphical Abstract:**
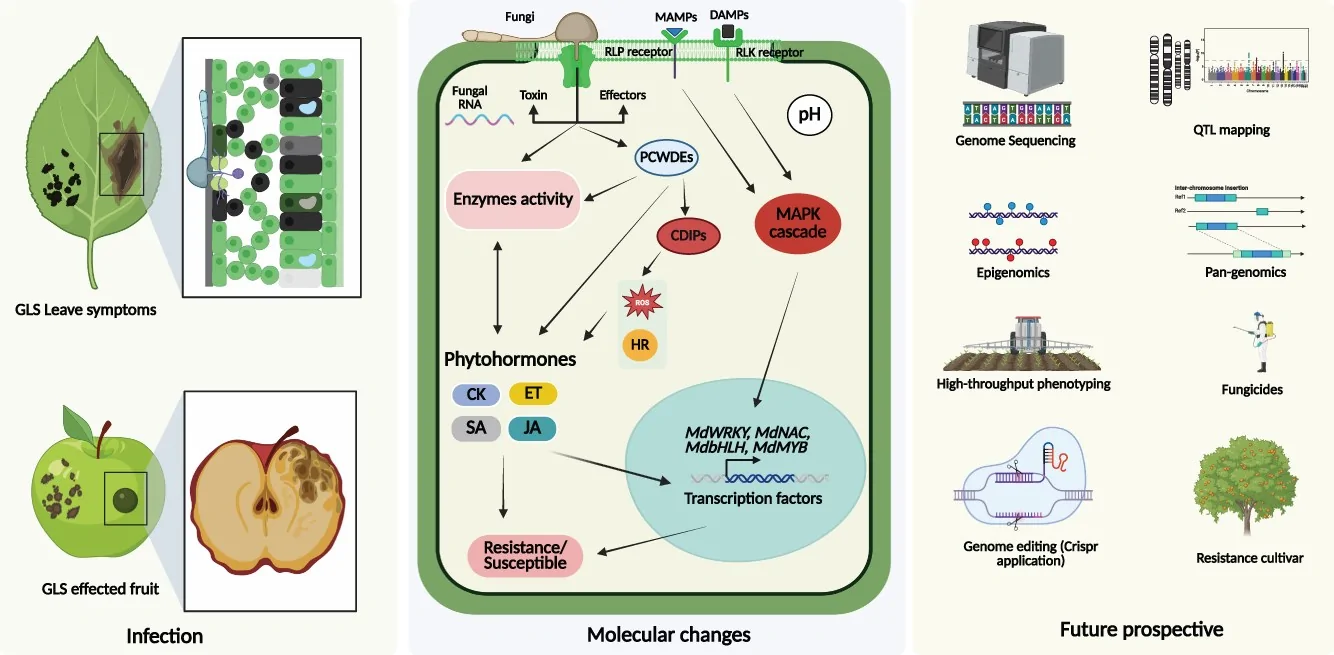

## Introduction

Apple (*Malus domestica*) is among the most extensively planted fruit tree species globally due to its significant output and commercial worth [1]. China is the leading global producer of apples, followed by Chile, the USA, France, and Italy [2]. However, apple production is severely constrained by various abiotic and biotic stresses that reduce growth, fruit quality and yield, resulting in considerable economic losses [3–5]. In natural environments, apple trees are continuously exposed to numerous pathogens, including viruses, bacteria, fungi, nematodes, and arthropods [6]. Among these, fungal diseases represent a significant threat to the sustainability of the apple sector in China. Glomerella leaf spot (GLS), induced by *Colletotrichum* spp. (Table 1), is a particularly destructive disease that affects foliage and, in some cases, extends to the fruit, significantly reducing yield and fruit quality [7]. This review summarizes current knowledge on GLS, including its epidemiology, pathogenic mechanism, host–pathogen interactions, plant defence responses, and current management strategies.

**Table 1:** *Colletotrichum* spp. responsible for GLS in apple.

**Disease type**	**Species group/complex**	**Species name**	**Geographical distribution**	**Ref**
GLS	*C. gloeosporioides complex*	*C. gloeosporioides*	Brazil, China, South Korea, USA	[49–51]
GLS		*C. fructicola*	Argentina, Brazil, China, France, Japan, South Korea, Uruguay, USA	[49, 52, 53]
GLS		*C. aenigmae*	China, Japan	[54]
GLS		*C. asianum*	China	[55]
GLS		*C. chrysophilum*	Brazil, Uruguay, USA	[56]
GLS	*C. acutatum* complex	*C. limetticola*	Brazil	
GLS	*C. boninense* complex	*C. karstii*	Brazil, Uruguay, USA	[24, 30]

The first report of bitter rot of apple in Great Britain was given by Berkeley in 1856 [8] and has since become a significant apple disease worldwide. Caused by the *Colletotrichum* complex, it primarily affects apple fruit. In contrast, GLS, which is also caused by *Colletotrichum* spp., mainly affects apple foliage. GLS was first observed in the southeastern United States in the cultivar ‘Golden Delicious’, where it was initially described as ‘necrotic leaf blotch’ [9]. Approximately a decade later, symptoms of GLS were first observed on both Gala and Golden Delicious cultivars in the State of Parana in Brazil during the late 1980s [10]. Subsequently, in 1998, GLS was reported to be a serious problem in two Gala orchards in eastern Tennessee, USA [11], and later in eastern China in 2012 [12]. Although bitter rot and GLS affect different plant tissues, both diseases are caused by related *Colletotrichum* spp. and are favored by warm and humid environmental conditions.

*Colletotrichum* spp. can affect several horticultural crops, such as almond [13], peach [14], grapes [15], strawberry [16], and blueberry, besides the major hosts apple and pear [17]. In mixed fruit orchards, the proximity of susceptible hosts may increase the risk of cross-infection [18]. Accurate identification of *Colletotrichum* spp. is challenging due to similar disease symptoms and morphological variability under different culture conditions [19–21]. Historically, bitter rot and GLS were attributed to *Colletotrichum gloeosporioides* teleomorph (*Glomerella cingulata*) and *Colletotrichum acutatum*, respectively [9, 22]. However, molecular phylogenetic analyses have revealed substantial diversity within these taxa, leading to recognition of the *C. acutatum* species complex and *C. gloeosporioides* species complex [23–25]. More recently, *Colletotrichum fructicola* was identified as a more predominant and infectious *Colletotrichum* sp. causing GLS in apple worldwide [26–29], and members of the *Colletotrichum boninense* species complex have also been associated with GLS in apple [30, 31].

The availability of advanced molecular tools, particularly multigene sequencing, has greatly improved species delimitation and epidemiological understanding of GLS pathogens [27, 28]. Given the economic impact of GLS, the development of resistant cultivars represents a sustainable management strategy. Conventional breeding has had limited success, although programs such as RRI (Purdue University, Rutgers University, Illinois (The University of Illinois) have released several scab-resistant cultivars carrying Rvi6(*Vf*) genes [32, 33]. Wild *Malus* species remain valuable genetic resources for disease resistance [34]. Recent advances in marker-assisted selection (MAS) and genomic selection (GS) have improved breeding efficiency and prediction accuracy [35, 36], and several countries are prioritizing cultivars combining disease resistance with high yield and fruit quality [33]. Emerging genome editing technologies, particularly CRISPR/Cas systems, provide new opportunities to investigate gene function and enhance resistance to fungal diseases [37–40].

Currently, fungicide application remains the primary method for controlling GLS in commercial orchards [41]. However, differences in fungicide sensitivity among *Colletotrichum* spp. and species complexes complicate disease management and may contribute to reduced control efficacy over time [42, 43]. These challenges highlight the importance of precise species-level identification and resistance monitoring to optimize fungicide selection and application programs. In addition to chemical control, integrated disease management strategies incorporating cultural practices, biocontrol agents, and plant-derived products are gaining attention as environmentally sustainable alternatives [44–47]. Furthermore, the release of a high-quality apple reference genome has facilitated rapid gene discovery, functional characterization, and improved understanding of host resistance mechanisms, thereby supporting the development of durable and science-based GLS management strategies [48].

Given the increasing prevalence and economic impact of GLS in major apple-producing regions, a comprehensive understanding of its pathogen diversity, host–pathogen interactions, and management strategies is essential for sustainable production. This review synthesizes current advances in molecular identification of *Colletotrichum* spp. associated with GLS, the mechanism of pathogenicity and plant defence responses, progress in resistance breeding and genome-based technologies, and integrated disease management approaches. By consolidating recent molecular, genomic, and applied research findings, this review aims to provide a scientific foundation to guide future studies and support the development of sustainable GLS management strategies.

## The pathogenesis of *Colletotrichum* spp. associated with GLS

**Figure 1:**
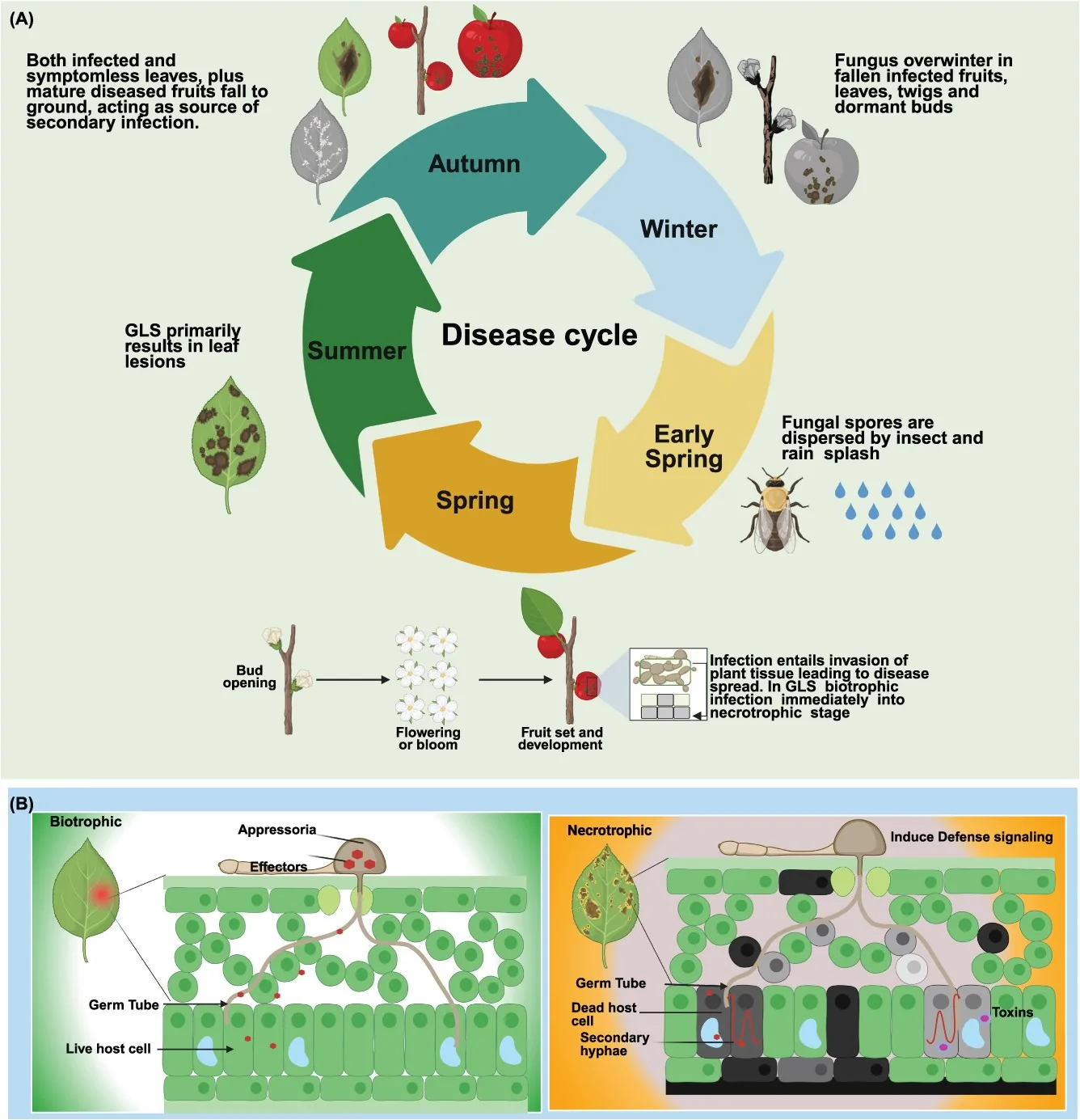
The cycle of *Colletotrichum* spp. responsible GLS in apple trees. (A) represents *Colletotrichum* spp. infection and survival cycle at various stages. (B) represents biotrophic and necrotrophic stages.

Fungal pathogenesis is a complex process that includes various mechanisms and pathways. Pathogenic species within the genus *Colletotrichum* are classified as hemibiotrophs, as their infection process involves two distinct phases: an initial biotrophic phase followed by a necrotrophic phase [57]. During the biotrophic phase, the fungus invades the host tissue, which allows it to establish itself by penetrating and colonizing the plant [58] (Fig. 1). However, as the infection progresses, *Colletotrichum* switches to the necrotrophic phase, actively killing host cells to extract nutrients, leading to tissue collapse and disease symptoms. This transition from biotrophic to necrotrophic phase is crucial for disease progression, enabling the pathogen to shift from active tissue colonization to symptom development within the host [59, 60]. These asymptomatic infections remain undetected during harvest but cause significant postharvest losses during storage [61]. *Colletotrichum fructicola* is a pathogen of major concern due to its severe impact on host plants during both the biotrophic and necrotrophic phases of infection [61, 62]. For example, a GLS pathogen, *C. fructicola*, causes suppression of the plant defence mechanisms during the biotrophic stage, which enables the pathogen to colonize the leaves successfully [27]. A study by Shang *et al*. [28] asserted that *C. fructicola* can cause GLS symptoms on apple leaves in <2 days. These results indicate that a short biotrophic stage is immediately followed by the necrotrophic phase. Numerous genes play a vital role in *Colletotrichum* pathogenicity and disease development.

Alkan *et al*. [63] reported that *pacC* in *C. gloeosporioides* modulates pH-responsive genes essential for pathogenicity, activating crucial enzymes in alkaline environments to facilitate host colonization. Liu *et al*. [64] identified that during GLS infection in apples, *CfSte12* is necessary for the formation of the appressorium, host penetration, effector regulation, and sexual reproduction. In *C. fructicola*, *CfMcm1* is an essential TF that controls infection, reproduction, and stress responses; *CfMcm1* mutant strains drastically lower virulence and regulate genes that control conidial development, appressorium formation, and sexual development [65]. Another TF, *CfCpmd1* in *C. fructicola*, governs the differentiation of plus and minus strains, perithecia formation, and conidiation. The *CfCpmd1* mutation impedes hyphal growth, sporulation, appressorium formation, and diminishes pathogenicity on apple leaves [66]. Additionally, Ren *et al*. [67] reported that ethylene augments the virulence of *C. gloeosporioides* by facilitating appressorium formation via G protein-coupled receptor (GPCR) and MAPK signaling pathways, with significant contributions from genes such as *CgSCD1, CgCP1, CgMK1*, and *CgSte11*. Further in-depth research is needed to elucidate the pathogenesis mechanism of GLS in apple caused by *Colletotrichum* spp.

Secondary metabolites also contribute significantly to pathogenicity of *C. fructicola*. A major gene cluster that encodes polyketide synthases (PKS) is conserved in multiple *Colletotrichum* spp. infecting apples, such as *C. gloeosporioides* and *C. fioriniae*. This cluster is specifically activated in the case of leaf infection and correlated with the synthesis of toxins and other molecules that induce disease progression [68]. Mitogen-activated protein kinase, *CfPMK1*, controls various processes of fungal growth and pathogenic infection, including the formation of appressoria and response to stresses [69].

### The role of *Colletotrichum* spp. effectors in pathogenesis

Beyond the general infection process, specific effector proteins play crucial roles in manipulating host immunity. Research on the secreted effectors of *Colletotrichum* spp. is growing and may enable the design of targeted strategies to limit critical effector functions, reducing host infection and disease progression [70]. Fungal effectors function as virulence factors, boosting pathogenicity by inhibiting or bypassing pattern-triggered immunity (PTI). Effectors are either common among *Colletotrichum* spp. or specific to certain species [71, 72]. Several effectors have been identified as playing a role in enhancing the virulence of *Colletotrichum* spp. (Table 1).

Shang *et al*. [27] identified an effector named CfEC92, a tiny secreted protein specific to *C. fructicola* that inhibits BAX-induced cell death in *Nicotiana benthamiana*. The *C. fructicola* ΔCfEC92 mutant reduced virulence on apple leaves and fruits. Tsushima *et al*. [71] mapped CEC3, an effector that has been demonstrated to promote nuclear enlargement and cell death in host plants, indicating its conserved function in enabling the necrotrophic phase among several *Colletotrichum* spp. Takahara *et al*. [77] identified that *C. higginsianum* effector ChEC91 causes cell death and plant defence responses in *N. benthamiana* and Arabidopsis, while its overexpression reduces fungal penetration in *Brassica rapa*; deletion studies showed that its C-terminal region is essential for activity.

However, *C. gloeosporioides* produces effector Sntf2, which reduces plant defences by preventing BAX-induced cell death, decreasing the formation of callose and H_2_O₂, and targeting the chloroplast Photosystem II (PSII) assembly factor *Mdycf39* [75]. Yang *et al*. [76] reported that CgNLP1 amplifies the virulence of *C. gloeosporioides* by interfering with the nuclear localization of the host defence regulator *HbMYB8* in pear, inhibiting salicylic acid–induced cell death and facilitating infection. Another study reported that CfRibo1 and CfRibo2 are secreted ribonucleases from *C. fructicola* that function as antimicrobial effectors, targeting pear-associated bacteria such as *Bacillus altitudinis* to facilitate fungal infection [74]. Hu *et al*. [72] mapped effectors Cf-ID1 and Cf-ID2 from *C. fructicola* that induce immune responses and cell death in tobacco, providing insights into its infection strategy in pecan. Han *et al*. [73] reported that *CfXyn11A* is a dual-function effector from *C. fructicola* that facilitates infection in pear while inducing immunity in tobacco; its activity is regulated by the pear apoplastic protein *PbXIP1,* which binds *CfXyn11A* to suppress its enzymatic activity and virulence. The studies above demonstrate that fungal effectors serve as dual-purpose tools: they directly inhibit plant growth while also exhibiting antagonistic effects against beneficial microbes.

Fungi that penetrate and destroy plant tissues to get nutrients are involved in fungal–plant interactions. Toxins that damage plant cells and effector proteins that suppress plant defences are released, which encourage infection and the spread of disease [73, 75, 76]. Numerous effector proteins from bacteria, oomycetes, and plant pathogenic fungi have been discovered (Table 2). *Colletotrichum fructicola* pathogenicity depends on specific secreted effector CfEC92, which is significantly expressed in appressoria during early apple infection and suppresses BAX-triggered cell death by modulating *PR1a*, *WRKY29*, and *MAPKKK3* expression [27]. According to Shang *et al*. [78], CfEC92 disrupts SA-mediated defence signaling and promotes fungal colonization by competitively blocking the interaction of the apple immune regulator *MdNIMIN2* with *MdNPR1*. A recent study revealed that CfEC92 binds ATP via a conserved Cx11NC motif, allowing structural modifications that improve its interaction with *MdNDPK2*. This connection facilitates the inhibition of host immunological responses by promoting *MdNDPK2* autophosphorylation and subsequent activation of *MdMPK3* [79]. Wang *et al*. [75] identified that C*. gloeosporioides’s* chloroplast-localized effector Sntf2 inhibits host immunity by interacting with the PSII assembly factor *Mdycf39* and lowering the build-up of callose and H_2_O₂. CfXyn11A from *C. fructicola* causes cellular apoptosis, Reactive Oxygen Species (ROS) build-up, and upregulates defence-related genes *WRKY7*, *Pti5*, and *CYP71D20* in *N. benthamiana*, through the activation of immune responses dependent on *BAK1* and *SOBIR1* [73]. Further studies are needed to find the interaction between fungal effector and plants.

**Table 2:** Identified effector proteins in *Colletotrichum*.

**Effector**	**Complex/species**	**Function**	**Localization**	**Reference**
CfXyn11A	*C. fructicola (gloeosporioides complex)*	Apoplastic xylanase; degrades cell wall, suppresses immunity	Apoplast	[73]
CfRibo1 / CfRibo2	*C. fructicola*	Secreted RNases; suppress competing microbes in phyllosphere	Apoplast	[74]
CfEC92	*C. fructicola*	Suppresses host cell death and immune response	Cytoplasm	[27]
Cf-ID1 / Cf-ID2	*C. fructicola*	Trigger HR-like cell death; early infection effectors	Cytoplasm /Nucleus	[72]
CEC3 homologs	*Multiple Colletotrichum species*	Induce nuclear expansion and programmed cell death	Host nucleus	[71]
Sntf2	*C. gloeosporioides*	Suppresses callose deposition & H₂O₂ accumulation; inhibits BAX-induced cell death	Chloroplasts	[75]
CgNLP1	*C. gloeosporioides*	Nep1-like necrosis/ethylene-inducing protein; promotes germination, virulence	Likely apoplastic/cell-surface	[76]
ChEC91	*C. higginsianum*	Induces necrosis and callose deposition	Extracellular/apoplastic	[77]

### Secondary metabolites and cell wall–degrading enzymes

In addition to effector proteins, GLS-associated *Colletotrichum* species secrete multiple cell wall-degrading enzymes (CWDEs) and metabolic virulence factors (Table 3). Key CWDEs, including polygalacturonases, pectate lyases, cellulases, xylanases, and cutinases, facilitate host penetration and progressive tissue maceration, particularly during the necrotrophic phase when pectin degradation accelerates lesion expansion and defoliation [80–83]. Furthermore, genomic analyses reveal abundant secondary metabolite biosynthetic clusters, notably PKS, nonribosomal peptide synthetases, and melanin-associated pathways, suggesting the production of bioactive compounds that may enhance colonization or modulate host immunity [84, 85]. Although GLS-specific metabolites remain largely uncharacterized, functional genomics and metabolomic approaches are needed to define their contribution to virulence.

**Table 3:** Potential of cell wall–degrading enzymes and secondary metabolites associated with virulence in GLS-related *Colletotrichum* species

**Virulence category**	**Enzymes**	**Function**	**Reference**
Cell wall–degrading enzymes	Polygalacturonases, pectate lyases	Involved in GLS lesion development	[80, 81]
	Cellulases		[80, 81]
	Xylanases		[80, 81]
	Cutinases		[80, 81]
	Proteases		[80, 81]
Secondary metabolites	PKS		[82]
	Nonribosomal peptide synthetases		[82]
	Melanin-associated pathways		[82]
	Phytotoxins		[82]

## Immune recognition and molecular response in apple

### Pathogen recognition

Apple initiates defence against *Colletotrichum* app. Through perception of conserved pathogen-associated molecular pattern and host-derived damage-associated molecular pattern, these signals are recognized by plasma member localized pattern recognition receptors (PRRs), triggering PTI [86]. PTI activation typically induces rapid ROS production, Ca^+^ influx, callose deposition, and activation of downstream kinases, forming a line of defence against fungal invasion in apple. Several receptors, like kinase (RLK) families, have been involved in fungal pathogen responsiveness. *MdCRK* genes show pathogen-responsive expression patterns and contain hormone-responsive promoter elements, suggesting roles in stress signaling [87]. Members of the SERK family contribute to immune regulation and hormone signaling [88], and *MdSERLK3* positively regulates resistance to *Valsai maili* via Ca^+^, H_2_O_2_, and callose signaling [89]. Wall-associated kinase-receptor-like kinase (WAK-RLK) genes (*MDP0000278283*, *MDP0000153539*, *MDP0000170906*, and *MDP0000251865*) have also been identified as potential contributors to fungal resistance [90].

Chitin represents the direct antifungal recognition pathway in plants. *MdCERK1* functions as a chitin-binding receptor and mediates chitin-induced immune response [91], while *MdCERKI-2* overexpression in tobacco enhanced antifungal defence in heterologous systems [92]. In addition to PTI, intracellular nucleotide-binding leucine-rich repeat receptor (NLR) proteins contribute to effector-triggered immunity. Several NLRs and NB-LRR proteins have been associated with resistance to *Alternaria* leaf spot, GLS, and other fungal diseases [93], with miRNA regulation (e.g. miRNA82 targeting *MdTNL1*) further modulating immune amplitudes [94, 95].

### MAPK cascades

Upon PRR activation, MAPK cascades immune signals to the nucleus. Apple MAPK cascades consist of MAPKKKs, MAPKKs, and MAPKs and function as central hubs connecting pathogen recognition to transcriptional responses [96]. The *MdMKK4*-*MdMPK3* cascade provides a mechanistic example of susceptibility regulation [97, 98]. *MdMPK3* phosphorylates *MdWRKY17*, promoting activation of the SA-degrading gene *MdDMR6* and reducing SA accumulation, thereby increasing susceptibility to *C. fructicola* [97]. This pathway demonstrates how MAPK signaling can modulate hormone homeostasis and influence disease outcomes.

Conversely, *MdMAPK6* has been reported to enhance resistance to fungal infection by promoting MAPK-mediated defence signaling [99], suggesting that the MAPK cascade may contribute both resistance and susceptibility-promoting branches depending on downstream targets. This finding highlights MAPK signaling as a decisive checkpoint that determines whether immune activation results in effective defence or enhanced susceptibility [96].

## Transcriptional reprogramming of GLS defence

**Figure 2:**
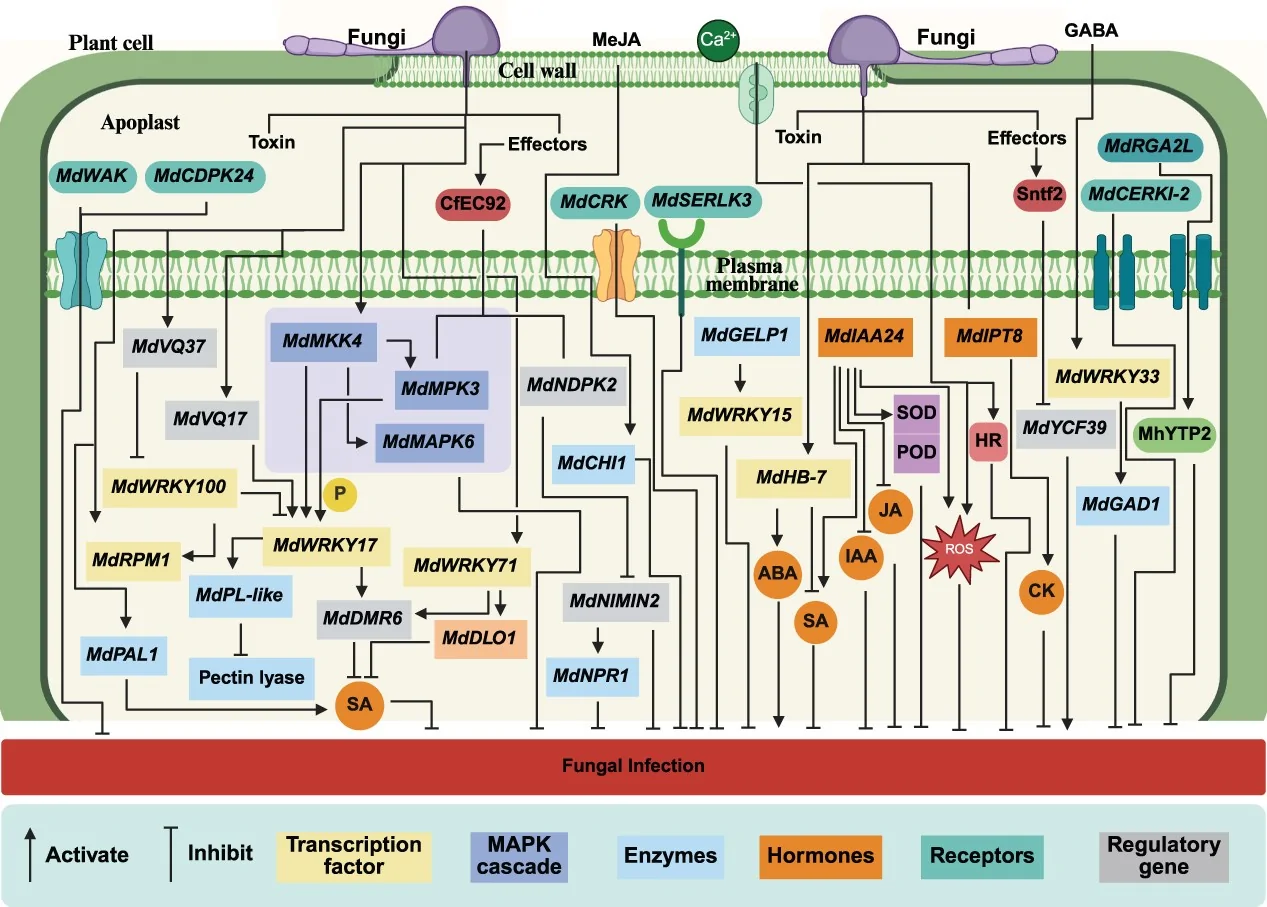
Integrated molecular network underlying apple response to GLS infection. This framework highlights the dynamic balance between SA-mediated resistance and JA/ET-associated modulation during GLS infection, emphasizing regulatory modes such as MdWRKY17/MdDMR6 as a critical determinant of susceptibility and resistance in apple.

Following MAPK activation, transcription factors (TFs) reprogram gene expression to modulate hormone pathways and defence outputs (Fig. 2). WRKY TFs form a central regulatory module in GLS responses. *MdWARKY100* enhances resistance to GLS by activating *MdPAL1* and promoting SA accumulation [100, 101]. In contrast, *MdWRKY17* and *MdWRKY71* act as susceptibility factors by activating SA catabolic genes *MdDMR6* and *MdDLO1* [102], reducing SA levels, and facilitating fungal infections. Regulatory genes such as *MdVQ37* and *MdVQ17* modulate WRKY activity, fine-tuning SA degradation and defence amplitude [97, 100]. Notably, *MdWRKY17* operates downstream of the *MdMKK4-MdMPK3* cascade, directly linking the MAPK cascade signaling to hormone reprogramming [98]. Li *et al*. reported that *MdWRKY33* directly activates *MdGAD1*, promoting aminobutyric acid biosynthesis; exogenous GABA treatment enhances antioxidant capacity and strengthens apple resistance to GLS. [103].

Other TF families integrate hormonal and metabolic outputs. MYB (*MdMYB73*) TFs regulate secondary metabolism and may influence flavonoid-mediated defence response [104]. NAC TFs contribute to stress regulation and proanthocyanidin biosynthesis, a compound associated with antifungal activity [105]. ERF TFs connect ethylene signaling to pathogenesis-related (PR) gene expression and lignification, contributing to structural defence reinforcement [106, 107].

HD-Zip and bHLH TFs further modulate hormone balance, oxidative stress responses, and disease resistance traits [108, 109]. Liu *et al*. [109] found that *MdHB-7* TF in apple diminishes resistance to GLS by lowering SA levels and defence enzyme activities while elevating abscisic acid content and hydrogen peroxide build-up. The overexpression of *MdHB-7* downregulates GLS resistance, while its silencing enhances defence responses. Although numerous TFs have been implicated, many studies rely on overexpression or other patho systems. Direct validation of Apple GLS infection remains essential.

## Hormone crosstalk during GLS infection

Phytohormones mediate growth defence trade-offs during pathogen attack (Fig. 2). SA plays a vital role in apple immunity, including systemic resistance [110]. However, GLS susceptibility is frequently associated with enhanced SA degradation by *MdDLO1* and *MdDMR6*, reducing defence intensity [111].

Jasmonic acid (JA) signaling influences defence responses and interacts antagonistically or synergistically with SA, depending on plant growth conditions. Auxin and cytokinin also modulate defence outcomes; *MdIAA24* overexpression increased SA levels and enhanced GLS resistance [112], while *MdIPT8* mediated cytokinin biosynthesis strengthened resistance to *C. gloeosporioides* [113]. Additionally, an ethylene-responsive factor further integrates ET signaling with defence gene activation and lignification. Despite strong evidence for SA’s importance, the dynamic interplay between SA, JA, and ET during different stages of infection remains poorly defined.

## Biochemical defence outputs

Hormone-driven transcriptional reprogramming culminates in activation of biochemical defences (Fig. 2). Enhanced phenylpropanoid metabolism contributes to lignin deposition, flavonoid accumulation, and antioxidant buffering. Enzymes such as PAL, C4H, 4CL, and CAD are differentially induced between resistant and susceptible cultivars [100]. Notably, *MdWRKY100* enhances apple resistance to GLS by directly activating MdPAL2, a key enzyme in the phenylpropanoid pathway, thereby promoting SA accumulation and downstream defence responses, whereas MdVQ37 negatively modulates thus pathway by inhibiting *MdWRKY100* activity, resulting in reduced MdPAL1 expression and SA levels.

In addition to phenylpropanoid metabolism, chitinases hydrolyse fungal cell wall components and contribute to apoplastic defence [114, 115]. *MdCHI1*, upregulated in auto tetraploid apple, enhances *C. gloeosporioides* resistance by boosting chitinase activity and is strongly induced by methyl jasmonate. GDSL lipases and related enzymes further suppress hyphal development and reinforce SA-mediated responses. Previously, Ji *et al*. reported that *GELP1* improves apple resistance to *C. gloeosporioides* by suppressing hyphal development and facilitating SA-mediated defence, exhibiting elevated expression in the resistant ‘Fuji’ variety and inducing *MdWRKY15* [116]. Together, these responses constitute the downstream response layer to GLS defence in apple. Although the precise interaction of enzymes and TFs during infection remains to be fully elucidated.

Collectively, Apple’s defence against GLS involves an integrated network linking pathogen recognition, MAPK signaling, transcriptional regulation, hormonal reprogramming, and biochemical defence response. PRR- and NLR-mediated receptors activate MAPK cascades that regulate TFs such as WRKY, MYB, and ERF, which subsequently modulate phytohormone pathways, particularly SA signaling and phenylpropanoid metabolism and antimicrobial enzymes. Notably, some signaling modules can function as either resistance or susceptibility regulators depending on downstream targets, as exemplified by the *MdMKK4*-*MdMPK3*-*MdWRKY17*-*MdDMR6* pathway that promotes SA degradation and increases GLS susceptibility. Despite these identifications, important knowledge gaps remain, including limited validation of candidate genes during *Colletotrichum* spp. infection in the apple. Incomplete understanding of SA-JA-ET crosstalk during different infection stages, and the largely unresolved targets of GLS-specific fungal effectors. Resolving these research gaps will be critical for advancing the development of GLS-resistant apple cultivars.

## Epigenetic modifications

Epigenetic regulation has emerged as an additional layer of immune control in the interaction between apple and *C. fructicola*. Recent evidence indicates that N6-methyladenosine (m^6^A) RNA methylation modulates disease resistance through m^6^A reader protein MhYTP2, which negatively regulates GLS resistance by binding to and destabilizing the transcript of *MdRGA2L*, an NBS-LLR resistance gene that promotes SA signaling. Elevated MhYTP2 activity reduces resistance to *C. fructicola*, whereas reduced activity enhances defence responses [117]. These findings highlight the roles of epigenetic regulation in SA-mediated immunity and suggest that *MdRGA2L* represents a promising target for resistance breeding. However, the broader contribution of DNA methylation and histone modification of GLS resistance remains largely unexplored.

## Advanced molecular techniques in GLS management

**Figure 3:**
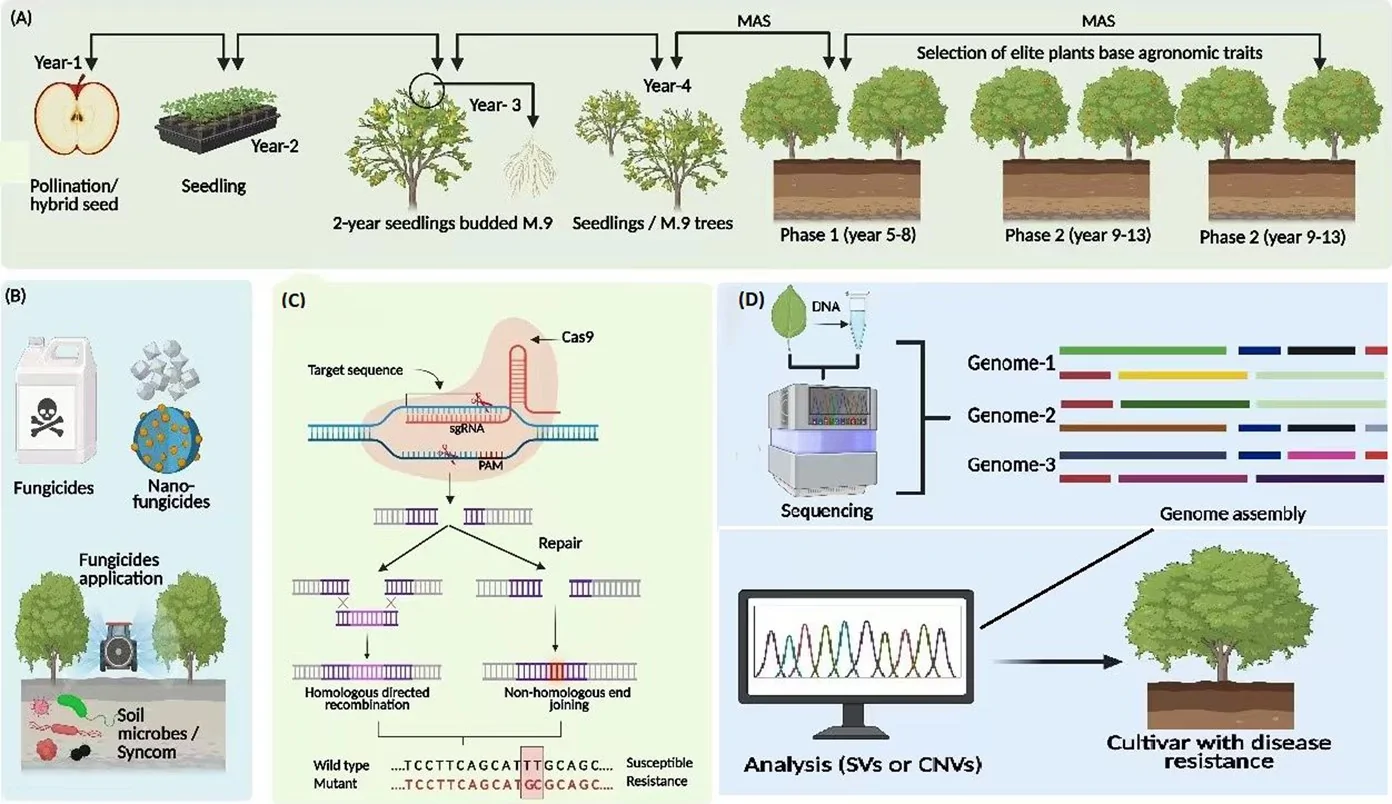
Application of different methods to develop GLS resistance apple line (A) Conventional breeding with MAS. (B) Application of fungicides, Nano-fungicides, and Syncom inoculum. (C) Crispr base genome editing (D) Genome sequencing and adoption of pan-genome in an apple breeding program.

Research into the correlation between fungal infections and apple defence aids the apple sector in developing efficient disease control strategies (Fig. 3). Traditionally, apples have been safeguarded against fungal infections through the application of chemical fungicides, essential oils (EOs), biological control agents, and agronomic practices [118–120]. The usefulness of preharvest spraying as a tool for disease prevention in apples has been extensively researched and is remains the main method for disease control. Before harvesting, the fruits are treated with functional coatings to improve quality and inhibit disease. Since those initial advancements, the prevalent application of synthetic fungicides, including triazole, prochloraz, benzimidazoles, benomyl, carbendazim, and strobilurins, has become normal [118, 119]. But excessive use of these generates resistance in the causal agent of GLS [121, 122]. Nanoparticles facilitate the regulated application of pesticides with great site specificity, hence minimizing collateral damage [123, 124]. Ternary Cu 2 SnS 3(CTS)/nano-TiO₂ composite coatings showed strong antifungal activity, achieving up to 100% inhibition of major postharvest pathogens by disrupting fungal cell (*C. gloeosporioides*) integrity [125].

In recent years, the utilization of plant EOs has emerged as a natural alternative to synthetic fungicides for the control of fruit fungal diseases [47]. A study reported that EOs derived from oregano, eucalyptus, fennel, sage, and rosemary were evaluated for their efficacy against *Penicillium expansum*, *C. gloeosporioides*, and *Botrytis cinerea* on apples. Oregano EO exhibited the most potent antifungal activity both *in vitro* and *in vivo*, markedly diminishing fungal proliferation Xylia *et al*. [126] reported that eucalyptus and rosemary EOs, together with their combination and eucalyptol, significantly reduced *P. expansum* infection in apples and pears (Fig. 3B) [127].

Artificial selection has produced a diverse array of cultivars exhibiting advantageous traits associated with crop productivity, growth time, and nutritional composition of fruit. Nonetheless, these strict breeding procedures have resulted in the loss of various traits, including resilience to biotic stresses, in cultivated genetic resources that were formerly present in the wild relatives of crops [128]. In response to the need to create resistant cultivars, researchers are investigating gene and genome-based breeding methodologies. A successful technique entails a combination of conventional breeding and MAS to incorporate specific genetic loci into new varieties [129, 130]. This approach immediately delineated the GLS resistance gene R (gls) to a 49-kb segment on apple chromosome 15 through genome re-sequencing and bulked segregant analysis, thereby pinpointing the essential SNP markers for MAS. Furthermore, it facilitates the creation of varieties with innovative gene combinations, a feat that is nearly impossible with traditional breeding methods, yet still necessitates a considerable time to attain the desired results (Fig. 3A) [130].

The split-marker method (SMM) is a genetic engineering technique used in fungi to replace a target gene with a selectable marker using two rounds of PCR procedures. It fuses ~1 kb flanking areas with incomplete marker sequences to generate two DNA cassettes that recombine in fungal protoplasts. Transformants are selected on a selective medium and evaluated for marker presence and the absence of the target gene [131]. Huang *et al*. [132] reported that *CgHox7* is crucial for appressorium development, penetration, and pathogenicity in *C. gloeosporioides.* Its targeted deletion by SMM validated its involvement in virulence, oxidative stress response, and cell wall integrity. According to Liang *et al*. [133], studies of *C. fructicola* reveal that plus and minus strains exhibit distinct cross- and self-fertilization behaviors, and that the *pmk1* gene is essential for mating line formation. These findings, often uncovered through Site-Directed Mutagenesis (SMM), are critical for understanding the genetic basis of fungal development and pathogenicity. Advanced breeding technologies are designed to enhance agricultural yield by creating climate-resilient, superior genotypes to address future global food security challenges (Fig. 3). These technologies, including gene editing (GE) utilizing zinc finger nucleases (ZFNs), transcription activator-like effector nucleases (TALENs), and CRISPR-Cas-associated nucleases, provide innovative approaches to improve the genetic traits of significant crops [38–40]. Compared to traditional methods, novel breeding techniques (NBTs) are more efficient and require less time to attain the desired outcomes. The Cas9-mediated double-strand break in DNA triggers repair mechanisms, including homology-directed recombination and nonhomologous end joining. Guide RNAs (gRNAs) direct Cas9, precisely locating target-specific regions [134].

CRISPR/Cas9 gene editing has been applied to apples since 2016 [37, 135]. Nishitani *et al*. successfully employed CRISPR/Cas9 to produce specific mutations in the apple *PDS* gene, resulting in albino plantlets and illustrating effective GE in apple. Pompili *et al*. employed CRISPR/Cas9 to knock out *MdDIPM4,* a susceptibility gene of apple, markedly reducing the incidence of fire blight disease induced by *Erwinia amylovora* [136].

Genome sequencing is vital to genetic research and effective evaluation of biological phenomena (Fig. 3D). Due to rapid breakthroughs in scientific study and biotechnology, genome sequencing has significantly developed, offering extensive insights into genomic structure and functional complexity [137]. A genome from one individual is inadequate to represent the complete genetic diversity of a species, particularly considering the diversification and modification of genomic organization associated with prolonged crop domestication. The pan-genome has emerged as a powerful approach to encapsulate the genetic diversity of crops and their wild relatives [138]. Apple domestication was driven by interspecific hybridization. Approximately 23% of the ‘Gala’ genome is hybrid, and selection sweeps from both progenitors influence critical features. Pan-genome and transcriptome analyses identified thousands of introgressed genes, with ~19% exhibiting allele-specific expression and many linked to fruit quality [139]. The *Malus* pan-genome disclosed significant copy number variations and structural variations affecting gene expression, fruit quality, and disease resistance. A 209 bp insertion in the MMK2 gene was demonstrated to generate a regulatory noncoding RNA that influences apple fruit colouring [103], A comprehensive framework for tracking genetic diversity, structural variation, and domestication history is provided by the pan-genome, which provides potent resources for future research in precision breeding, trait enhancement, and sustainable agricultural development.

## Conclusion

GLS has emerged as one of the most destructive foliar diseases of apple, significantly compromising fruit quality and fruit yield in major production regions around the world. Accumulating evidence supports a hierarchical immune framework in which pathogen recognition by PRR and NLR proteins activates MAPK cascades, leading to TF-mediated modulation of hormonal networks, particularly SA homeostasis, and culminating in biochemical defence involving phenylpropanoid metabolism, PR protein modulation, and lignification. Among various plant responses, the dynamic regulation of SA biosynthesis and degradation, controlled by MAPK-WRKY interaction, appears to represent a central checkpoint determining resistance versus susceptibility outcomes.

Despite substantial progress, several fundamental challenges remain unclear. First, GLS-specific virulence determinants and effector of *Colletotrichum* spp. remain poorly characterized, limiting our understanding of pathogen adaption and host specialization. Second, although SA is clearly important in resistance, the antagonistic and synergistic interactions between JA and ET during different infection stages remains insufficiently resolved. Third, many candidate resistance genes have been validated primarily under controlled conditions; their efficacy across diverse germplasm backgrounds and fluctuating environments remains largely unknown. Finally, pathogen evolution and population diversity raise concerns regarding the long-term stability of single gene resistance strategies.

**Figure 4:**
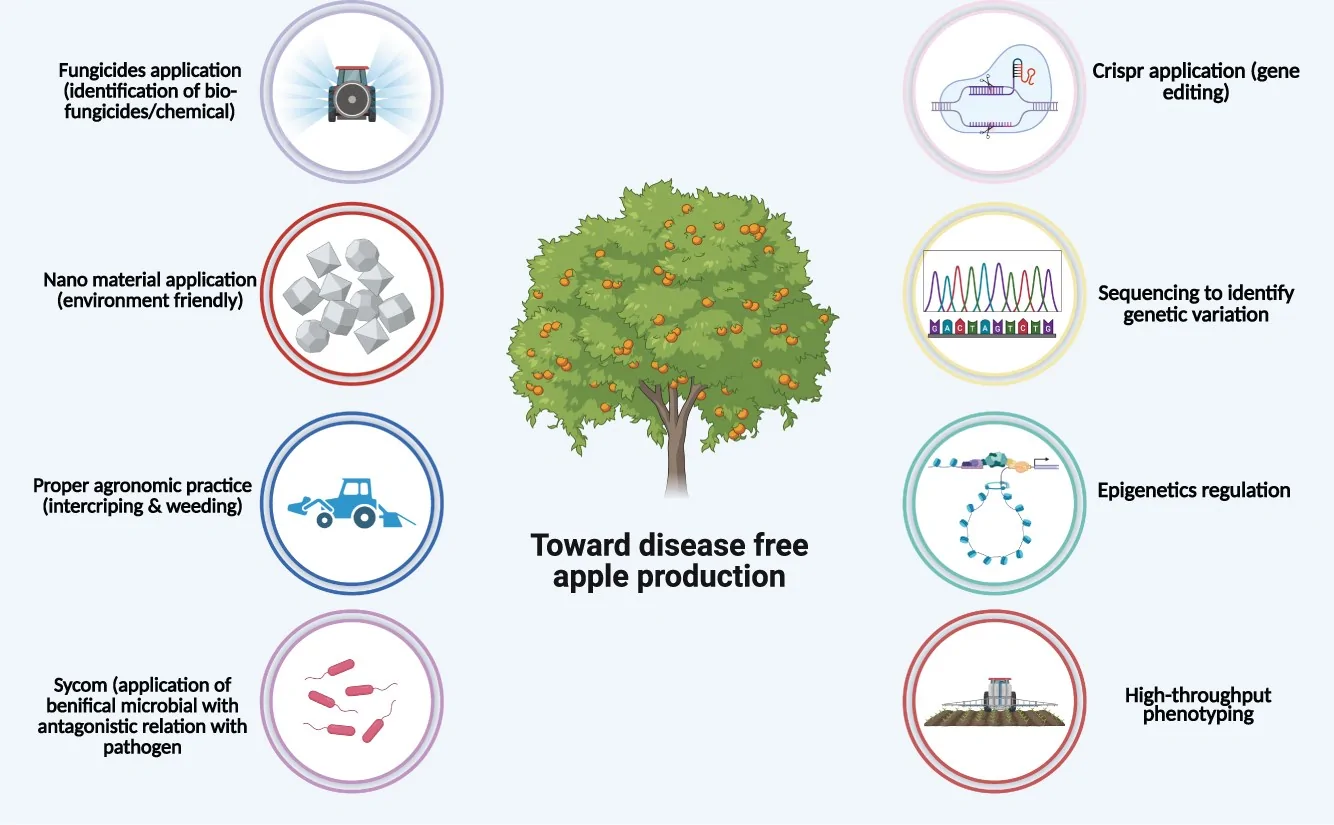
Comprehensive application of fast-forward various tools for disease smart apple plants.

Future GLS management must therefore integrate mechanistic molecular insights with precision breeding and sustainable agronomic strategies (Fig. 4). High-throughput sequencing and pan-genomics analyses will enable identification of resistance loci across diverse germplasm. CRISPR/Cas-mediated genome editing offers opportunities to modify susceptible genes and fine-tune immune regulatory pathways. Epigenetic regulation and transcriptomic plasticity may further contribute to adaptive resistance. Concurrently, integration of disease management approaches including optimized fungicides and nano-formulations. Beneficial microbial consortia, and improved orchard practices remain essential to reduce pathogen pressure while resistant cultivars are being developed. Achieving durable, disease-resistant apple production requires an integrated approach that connects pathogen biology, host immunity, and advanced genomic and breeding technologies. Despite advances, several fundamental knowledge gaps still limit the development of GLS resistance cultivars.

Future research should focus on identifying GLS-specific effector receptor interactions and clarifying hormone crosstalk during infection through integrated multi-omics approaches. CRISPR/Cas-based gene validation of resistance and susceptible cultivars, combined with multi-location evaluation, will be essential for immune response. In addition, pan-genomic exploration of diverse germplasm and integration of GS with high-throughput phenotyping will accelerate development of resilient apple cultivars.

## Recommendations

Presented below are concise recommendations for future research GLS:

- Effector function: Identify principal effectors (e.g. CfEC92, CfXyn11A) and their host targets to elucidate mechanisms of immune suppression.
- Host–pathogen interactions: Understanding genetic bases signaling pathways and defensive responses influenced by fungal infection.
  Resistance strategies
  Use of NBTs or genetic resistance approaches and explore hormone- or pH-mediated infection cues in apple.
